# About millets and beans, words and genes

**DOI:** 10.1017/ehs.2020.33

**Published:** 2020-06-15

**Authors:** Martine Robbeets, Chuan-Chao Wang

**Affiliations:** 1 Eurasia3angle Research group, Max Planck Institute for the Science of Human History, Jena, Germany; 2 Department of Anthropology and Ethnology, Institute of Anthropology, National Institute for Data Science in Health and Medicine, and School of Life Sciences, Xiamen University, Xiamen 361005, China

**Keywords:** Transeurasian, triangulation, genetics, linguistics, archaeology, Neolithic, millet agriculture

## Abstract

In this special collection, we address the origin and dispersal of the Transeurasian languages, i.e. Japonic, Koreanic, Tungusic, Mongolic and Turkic, from an interdisciplinary perspective. Our key objective is to effectively synthesize linguistic, archaeological and genetic evidence in a single approach, for which we use the term ‘triangulation’. The 10 articles collected in this volume contribute to the question of whether and to what extent the early spread of Transeurasian languages was driven by agriculture in general, and by economic reliance on millet cultivation in particular.

## Graphical abstract

**Figure:**
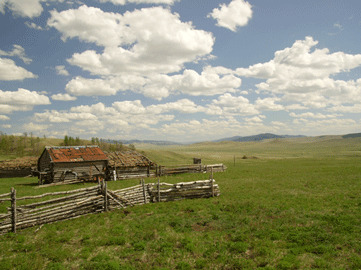

**Media summary:** In this special collection, we address the origin and dispersal of the Transeurasian languages, by synthesizing linguistic, archaeological and genetic evidence in a single approach.

The origin and dispersal of the Transeurasian languages is among the most fervently disputed issues in historical comparative linguistics. In this special collection, we address this topic from an interdisciplinary perspective. Our key objective is to effectively synthesize linguistic, archaeological and genetic evidence in a single approach, for which Bellwood (2002: 23–25) introduced the term ‘triangulation’, generalizing Kirch and Green (2001)'s earlier application of the term to Polynesian language and archaeology.

One of the main questions that inspired the ‘millets and beans, words and genes’ in our title is whether and to what extent the early spread of Transeurasian languages was driven by agriculture in general, and by economic reliance on millet cultivation in particular. The beans have not only been added to the title for their rhyming character, but represent the relevance of legumes, which should not be seen as a mere addition for a balanced diet, but as an essential element for the development of a successful agricultural system, for instance through crop rotation.

To answer our question, we brought together experts in linguistics, archaeology and genetics and motivated them to leave the comfort zone of their own discipline. In order to enhance interdisciplinary collaboration, we tried out a ‘tandem’ format by inviting geneticists, linguists and archaeologists to a conference in Jena, on condition that they could extend the invitation to at least one co-author from outside their own discipline. The conference, which took place from 8 to 11 January 2019, was organized by Martine Robbeets and the eurasia3angle team and generously funded by a grant from the European Research Council (ERC). Well attended by nearly 100 participants, it generated new insights and stimulated discussion. Therefore, we decided to publish a selection of papers resulting from our conference.

Elaborating on the conference theme, the purpose of the present special collection is to examine whether the Transeurasian languages owe their distribution, at least in part, to the demographic, cultural and linguistic processes which accompanied the dispersal of millet cultivation in North and East Asia.

## The Transeurasian languages

**Figure 1.**
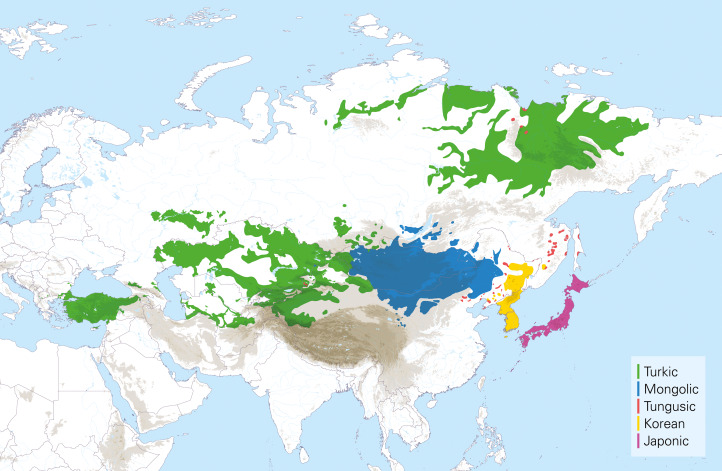
The distribution of the Transeurasian languages.

Although traditionally referred to as ‘Altaic languages,’ Johanson and Robbeets (2010: 1‒2) coined the term ‘Transeurasian’ to refer to a large group of geographically adjacent languages, stretching from the Pacific in the east to the Baltic, the Black Sea and the Mediterranean in the west. This grouping includes up to five uncontroversial linguistic families: Japonic, Koreanic, Tungusic, Mongolic and Turkic. It is distinguished from the more traditional term ‘Altaic’, which we here reserve for the linguistic grouping consisting of Tungusic, Mongolic and Turkic languages only. Figure 1 displays the distribution of the Transeurasian languages.

The Turkic language family consists of about 35 closely related Turkic languages and dialects spoken over a wide area of the Eurasian continent, including some parts of Europe, Asia Minor, Central Asia and Siberia. The earliest clearly documented stage is the language of the Eastern Old Turkic inscriptions of the eighth century AD in Mongolia's Orkhon valley. In their contribution to this collection, Alexander Savelyev and Choongwon Jeong (2020) discuss nomadic groups associated with ancient varieties of Turkic that ruled over the Eurasian steppe several centuries before the early inscriptions, such as Xiongnu, Hun and Avar groups. Junzo Uchiyama et al. (2020) take us back to the spread of the ancestral Proto-Turkic-speaking community around 2000 years ago.

The Mongolic language family consists of about 15 closely related languages, extending over Central and Northeast Asia. All contemporary Mongolic languages can be traced back to the language spoken by Chinggis Khan, the founder of the Mongol empire (1206–1308). The earliest documented stage is Middle Mongolian, with the most well-known text being the ‘Secret History of the Mongols’, originally compiled in the mid-thirteenth century, but preserved in a modified seventeenth-century copy. Proto-Mongolic, the language ancestral to the contemporary Mongolic languages, is thought to have split from Proto-Khitanic in the second century AD. Among the Khitanic languages are the now-extinct languages of the Tabgach of the Northern Wei Dynasty (386–550) and the Khitan of the Liao dynasty (916–1125). Alexander Savelyev and Choongwon Jeong (2020) further suggest that the Rouran people, who ruled Mongolia and adjacent areas between 300 and 550 AD were speakers of a sister language of Mongolic.

The Tungusic family comprises about 15 languages distributed over Manchuria and Siberia. Since written materials in Jurchen, the now-extinct language of the Jin dynasty (1115–1234), are only partially deciphered, the earliest well-documented stage is Manchu, the official language of the Qing dynasty (1644‒1911). As discussed in the contribution by Chuan-Chao Wang and Martine Robbeets (2020), all contemporary and ancient varieties of Tungusic descend from a common ancestral language, which we call ‘Proto-Tungusic’.

Today there is only a single Korean language, but as explained in the contribution by Jangsuk Kim and Jinho Park (2020), in the past various Koreanic varieties coexisted with Japonic languages on the Korean peninsula. Evidence for their presence is provided by, among others, a corpus of toponyms in Korean historical sources. Among the now-extinct Koreanic languages we find the languages of Paekche, Kaya and Silla, spoken before the linguistic unification of the peninsula in 668 AD. Some fragments of writing go back to before that time, but a systematic and accurate documentation of the Korean language started only with the Hangŭl texts in the fifteenth century.

We can distinguish between Japanic languages on the one hand and Japonic languages on the other. The term ‘Japanic’ is used in reference to the historical varieties of the Japanese language spoken on the Korean Peninsula in addition to those spoken on the Japanese islands. In contrast, the label ‘Japonic’ is restricted to the insular variety, the language family composed of Mainland Japanese and the Ryukyuan languages. Several articles in this special edition, such as those by Mark Hudson et al. (2020), Elisabeth de Boer et al. (2020) and Gyaneshwer Chaubey & George van Driem (2020), deal with the advent and spread of Proto-Japonic. Although the authors disagree on the exact timing, they all agree that Japonic entered the Japanese islands via the southern tip of the Korean peninsula, with the ancestral speakers settling in northern Kyushu and eventually spreading to the rest of the Japanese islands. The earliest clearly documented stage of Japonic is Old Japanese, dating back to the eighth century AD.

Hence, the Transeurasian languages form a vast linguistic continuum that crosses the physical boundaries between Europe and Asia. Although most linguists would agree that these languages are historically related, they disagree on the precise nature of this relationship: are all similarities induced by borrowing, i.e. horizontal transmission, or are some residues of inheritance, i.e. vertical transmission? Scholars who take an areal approach – so-called ‘diffusionists’– admit that the Transeurasian languages share many common linguistic elements and features, but they maintain that these are better accounted for by an interplay of borrowing, universal principles in linguistic structuring and coincidence, than by common descent. In contrast, scholars who take a genealogical approach – so-called ‘retentionists’– admit that the Transeurasian languages have been subject to extensive mutual contact throughout their histories, but they maintain that not all similarities are the result of borrowing, universals or chance. They argue that there is a limited core of similarities for which the most sensible linguistic explanation is inheritance. Although most contributions in our special edition take a neutral attitude with regard to the deeper affinity of the Transeurasian languages, four contributions, notably those by Cui et al. (2020), Nelson and colleagues (2020), Wang and Robbeets (2020) and Chaubey and van Driem (2020), accept the affiliation hypothesis as a point of departure for their research.

## Farming language dispersal

The ‘Farming/Language Dispersal Hypothesis’, a proposal originally advanced by Renfrew (1987), Bellwood and Renfrew (2002), Diamond and Bellwood (2003) and Bellwood (2005, 2011), posits that many of the world's major language families owe their primary dispersals to the adoption of agriculture by their early speakers. Since farming can unquestionably support far greater population densities than hunting and gathering, the basic logic behind this hypothesis is that population growth steadily pushed early farmers and their language into wider territories.

### Three mechanisms

The mechanisms involved in this process are three-fold and mutually compatible; there is room not only for migration, but also for adoption and diffusion. The most simplistic scenario is migration without interaction, when preexisting hunter–gatherer populations are pushed to the peripheries, leaving their original habitats open to occupation by incoming farmers. From a linguistic viewpoint, this would lead to language displacement: the hunter–gatherer language is expelled to marginal areas while the incoming farmers’ language fills the void.

In reality, however, migration does not take place in a vacuum: migrants will only rarely fill demographically and linguistically empty spots. Instead, local hunter–gatherer populations may stay and play a more active role, choosing to either adopt farming from the incoming migrants or not. Language shift, a change whereby speakers abandon their previous native language in favour of a target language, is a common linguistic by-product of adoption. In this process, the abandoned language becomes a substratum, i.e. an underlying historical stratum. Substratum interference, also called ‘imposition’ (Van Coetsem, 2000; Johanson, 2002; Winford, 2013), can be established when there are indications that the substratum influenced the target language as part of the process of language shift. According to Thomason and Kaufman (1988: 38), substratum interference results from ‘imperfective group learning during a process of language shift’ and will more easily affect language structure than concrete words. Nevertheless, the newly adopted language is genealogically related to the ancestral language of the migrants.

Simultaneously, part of the indigenous hunter–gatherer populations may prefer to maintain their local lifestyles, subsistence strategies and languages, but nevertheless interact with the migrant farmers. This will lead to various cultural exchanges in the hunter–gatherer–farmer border zones, the linguistic outcome of which is expected to be borrowing, starting with the diffusion of cultural lexicon. In this collection, Cui et al. (2020) and Wang and Robbeets (2020) regard Nivkh as descending from an ancient local linguistic lineage of hunter–gatherers that became isolated when the languages of Tungusic farmers expanded. While adoption led to Nivkh substratum interference in Tungusic, diffusion resulted in ancient borrowing between both languages. Similarly, Hudson et al. (2020) posit a multicultural context of interaction between Jomon and Yayoi populations on the Japanese islands, involving cultural exchange and language contact between speakers of ancient varieties of Ainu and Japonic.

### Indo-European is not a schoolbook example

Although the idea of farming/language dispersal was initially developed with the case of Indo-European (Renfrew, 1987) and Austronesian (Bellwood, 1987, 1991) in mind, the Indo-European language family does not represent a textbook example of the hypothesis in the way Austronesian does. Uncertainty about agriculture-driven expansion marks the debate between the Anatolian hypothesis, which suggests that farmers migrated out of the Middle East around 7000 BC, and the Steppe hypothesis, which suggests that herders migrated out of the Eurasian steppe around 4000 BC. The former hypothesis, which is in accordance with Renfrew's (1987) view of farming/language dispersal, is supported by certain ‘quantitative’ linguists using Bayesian phylogenetic methods (Gray & Atkinson, 2003), while ‘qualitative’ linguists, relying on classical historical comparative reconstruction, tend to support the latter hypothesis (Mallory, 1997; Comrie, 2002; Anthony & Ringe, 2015; Iversen & Kroonen, 2017: 513–516).

Whereas quantitative and qualitative linguistic research seem to point in different directions for Indo-European, both approaches reinforce each other in postulating agricultural spread for numerous language families in Asia, such as Austronesian (Blust, 1995, 2013; Pawley, 2002; Bellwood & Dizon, 2008; Gray et al., 2009), Sino-Tibetan (Sagart et al., 2019; Zhang et al., 2019), Tai-Kadai (Ostapirat, 2005: 128), Austroasiatic (Higham, 2002; Diffloth, 2005; Sidwell and Blench, 2011; Sagart, 2011) and even Transeurasian (Robbeets and Bouckaert, 2018; Robbeets, 2020). These language families probably represent much better examples of farming/language dispersal than does the Indo-European case.

### Language dispersal without farming, farming dispersal without language

By positing that ‘many’ of the world's major language families spread with agriculture, the Farming/Language Dispersal Hypothesis does not assert that ‘all’ of them did. This is obvious because some widely spread language families such as Pama–Nyungan in Australia or Eskimo–Aleut in North America never developed farming in the first place. Moreover, we find widespread families for which agricultural vocabulary can be confidently reconstructed, but where it remains unclear whether agriculture is indeed the main driving force behind the spread. This is, for instance, the case for Indo-European as discussed above, the Quechuan and Aymaran languages (Emlen & Adelaar, 2017) and even the initial Bantu expansion (Bostoen & Koni Muluwa, 2017). Nevertheless, even if not all language dispersal is caused by farming, farming/language dispersal remains a viable hypothesis for many language families across the world.

Similarly, not all farming dispersal is expected to cause language shift. There are several examples of crop dispersal resulting in the borrowing of the crop name and other agricultural vocabulary associated with it without causing language shift. This is the case for several documented farming dispersals: the pre-Columbian diffusion of the sweet potato from the Andean region to the South Pacific and associated borrowings from Quechuan and Aymara into Polynesia (Adelaar & Muysken, 2004: 41); the dispersal of barley and wheat agriculture from the Fertile Crescent through the Near East to China diffusing crop names into Indo-European, Old Chinese and individual Transeurasian languages (Blažek, 2016; Robbeets, 2017a; Kümmel, 2017); the dispersal of taro cultivation from Southeast Asia to New Guinea, inducing various borrowings across the languages of New Guinea (Schapper, 2017); or the spread of rice agriculture to the Korean peninsula with borrowing from Austronesian or Sinitic into Japanic (Robbeets, 2017b) and from there into Koreanic (Vovin, 2015). Remarkably, in most of these examples the introduction of the particular crop constitutes the innovation of a pre-existing agricultural package rather than a transition from foraging to agriculture. The linguistic outcome of farming dispersal thus appears, at least in part, to be determined by how revolutionary the newly imported agriculture is with respect to the local population's subsistence strategies. Additions to pre-existing subsistence strategies are expected to induce linguistic borrowing, while life-changing innovations are more likely to lead to language dispersal and shift.

In addition, crop-specific factors, such as productivity, may also influence the linguistic outcome of agricultural dispersal. Observing the high productivity of rice *vis-à-vis* millets, archaeobotanists argue that wet rice leads to ‘packing in’ while millet encourages ‘spreading out’ (Fuller and Qin, 2009; Stevens and Fuller, 2017). More specifically, wet rice cultivation can absorb population increase through intensification of land use, while the increased production of millet tends to occur through the agricultural colonisation of new land. Therefore, rice tends to be spread more easily through cultural diffusion, while millets are more frequently spread by population migration. The two scenarios are expected to yield different linguistic outcomes: in the case of rice with diffusion, borrowing is the expected outcome, while in the case of millet with migration, language dispersal and shift are frequently observed.

### A more general subsistence model

We can further relativize the importance of agriculture as a trigger for language spread by regarding farming/language dispersals as just one instantiation of the subsistence/demography model, originally proposed by Renfrew (1987: 123–131). Migrants importing a new subsistence strategy unknown to the local population, or innovating a pre-existing subsistence strategy so that it becomes significantly more effective or productive, will convince the local population to abandon their language and shift to the new target language. They conquer not by force, but by efficiency. The key issue is an advantage in subsistence strategy and thus expansive potential – be it related to foraging, farming or pastoralism – that eventually makes the incoming population demographically more successful than the local one, an argument first made by Bettinger and Baumhof (1982) for the spread of Numic-speaking people in the American Southwest.

We already noted that some well-spread families such as Eskimo–Aleut have no farming, but subsistence nevertheless played a role in their development, in that the language spoken by the population gaining access to the food resources replaced the pre-existing language spoken by the population losing access (Berge, 2017). Other families, such as Indo-European and – as discussed by Uchiyama et al. (2020) in this collection – Turkic, may have been familiar with farming but their spread was caused by food surpluses and mobility associated with horse-riding pastoralism.

Moreover, under certain conditions, adopting mixed subsistence strategies may be more efficient than exclusively practising agriculture. Especially in regions with various ecological environments and climatic conditions, mixed subsistence may increase access to diverse food resources. Uchiyama et al. (2020) in this collection point out that the Altai–Amur–Japan region, where the major Transeurasian linguistic dispersals took place, packs a variety of habitats, such as different kinds of forests, grasslands, and freshwater and maritime environments, into a relatively small area. In addition, over the last 8,000 years, the climate in this region has been marked by a particular variability (Jia et al., 2017), which has repeatedly disrupted traditional resource bases. Adding agriculture to a subsistence package that already consisted of other strategies such as hunting, gathering and fishing helped the Transeurasian speech communities to expand into different natural environments and adapt to changing climatic conditions. The relevance of mixed subsistence strategies, including agriculture, is a common thread throughout our collection. See the contributions by Savelyev and Jeong (2020), Robbeets and Wang, Kim and Park (2020) and Uchiyama et al. (2020).

## The ancient DNA revolution

With the advent of next-generation sequencing technology 10 years ago, ancient DNA research has opened a genomic era for the study of prehistoric human migration. Recently, it has been widely used to assess the Farming/Language Dispersal Hypothesis for several major language families, especially Indo-European. So far, fine-scale ancient DNA sampling of West Eurasia has documented the migration of the first farmers from the Near East to Europe during the Early Neolithic in addition to a later major turnover, 4,500 years ago, whereby steppe pastoralists associated with the Yamnaya culture massively migrated and replaced about 75% of the ancestry of central Europeans (Haak et al., 2015; Allentoft et al., 2015; Lazaridis et al., 2016). About 500–1,000 years later, these steppe pastoralists migrated to South Asia, contributing up to 30% of the ancestry of modern groups there and supporting the Steppe hypothesis for the spread of Indo-European languages (Narasimhan et al., 2019).

In spite of the fact that ancient DNA studies to date have been highly focused on Europe, recent progress in East and Southeast Asia and Oceania has shed valuable light on the history of Austronesian, Austroasiatic and Sino-Tibetan populations. Ancient samples from Vanuatu and Tonga dating to about 2,900–2,600 years ago associated with the Lapita culture are genetically very similar to present-day Austronesian speakers in Taiwan, reflecting the spread of Austronesian languages, probably driven by agriculture, from Taiwan to Southeast Asia and Oceania (Skoglund et al., 2016).

This farming/language dispersal then triggered a population turnover in remote Oceania shortly after the initial settlement of Lapita people. Papuan ancestry had arrived in Vanuatu by around 2500 BP, and was subsequently diluted through admixture but remains at least at 80–90% on most islands (Lipson et al., 2018a; Posth et al., 2018). However, the incoming Papuan languages did not replace Austronesian languages. The early farmers from Man Bac in Vietnam were suggested to be a mixture of southern Chinese agriculturalists and hunter–gatherers related to Andamanese, and this mixture profile is also characteristic of present-day Austroasiatic speakers in a vast region from mainland Southeast Asia to as far south as Indonesia, providing evidence for an agriculture-initiated spread of Austroasiatic languages (Lipson et al., 2018b; McColl et al., 2018). The Neolithic farmers of the Wuzhuangguoliang sites from the middle Yellow River Basin were genetically closer to both Tibetan and Han Chinese, which is consistent with the farming-related northern-origin hypothesis for the initial expansion of Sino-Tibetan languages (Wang et al., 2020).

Ancient genomes from Mongolia, Northeast China and the Russian Far East, spanning more than 8,000 years, document a genetic lineage that may be associated with speakers of Transeurasian languages (Siska et al., 2017; Wang et al., 2020; Jeong et al., 2020; Yang et al., 2020; Ning et al., 2020). This genetic lineage is best represented by Neolithic samples from Inner Mongolia, Mongolia and the Russian Far East, but also contributed to later groups from the Late Neolithic to present-day Tungusic, Mongolic and Turkic speakers in those regions, documenting an expansive initial spread and long-time persistence of that type of ancestry. In this collection, Wang and Robbeets (2020) combine archaeolinguistic evidence with ancient genomes to identify the region around Lake Khanka in the Russian Far East as the most plausible homeland for the ancestral speakers of Tungusic. Cui and colleagues take genetic continuity as supporting evidence for the Farming/Language Dispersal Hypothesis that the expansion of the Transeurasian language family was related to the agricultural development and expansion of the Hongshan culture in the Amur River Basin. With regard to Japan, the Farming/Language Dispersal Hypothesis is reviewed in this collection from genetic, archaeological and linguistic perspectives by de Boer et al. (2020) and Hudson et al. (2020). Present-day Japanese are suggested to be an admixture of hunting–gathering Jōmon and farming Yayoi populations. Ancient genomes of the Jomon suggest that they were genetically a deeply diverged lineage compared with other East Asians, but without available ancient DNA from Yayoi people, it remains unclear how the Jomon peoples interacted with migrant Yayoi agriculturalists and whether and to what extent the Yayoi genetically derived from the ancestry represented by the Neolithic samples from Inner Mongolia, Mongolia and the Russian Far East.

## Organization and argumentation

The first contribution to our special collection was led by Sarah Nelson, Emeritus Professor of Anthropology and Distinguished University Professor at the University of Denver, who passed away on 27 April 2020, hardly a month after finalizing her article. Sarah was a world-recognized expert on the archeology of Korea and Northeast China, a feminist who altered the discussion of gender in archeology, the editor of 13 volumes and the author of three novels, nine scholarly books and over 150 articles. She designed the archaeological part of the ERC eurasia3angle project that underlies this special collection and continued to provide active support until her last days. Encouraging us to cross boundaries and to integrate different disciplines, Sarah introduced us to many of the esteemed archaeologists contributing here.

Together with Irina Zhushchikhovskaya, Tao Li and Mark Hudson, archaeologists specializing in the Russian Far East, Northeast China and Japan, respectively, Sarah Nelson and Martine Robbeets trace population movements in ancient East Asia through the linguistics and archaeology of textile production. They test the hypothesis that Transeurasian language dispersal was initially agriculture-driven by investigating words and material evidence beyond agriculture. This approach comes from the expectation that, when agriculture drives speech communities to migrate, other aspects of material culture will move as well. To this end, they examine parallels between early dispersal patterns of textile technology, farming, language and people, an analysis that has not yet been undertaken for North and East Asia. They show a correlation between the use of agriculture, textile production and the words reflecting these activities in the ancient cultures and languages of the Transeurasian-speaking region. From this, they infer that the transition to more sophisticated textile technology can be associated not only with the adoption of millet agriculture, but also with the spread of the languages of the Transeurasian family. At the same time, their results provide an answer, at least in part, to the reservations made by Kim and Park (2020) in this collection about the lack of elements of Northeast Chinese material culture introduced together with millet agriculture. Moreover, their research provides indirect support for the Language/Farming Dispersal Hypothesis, which posits that language expansion from the Neolithic onwards was often associated with agricultural colonization. From a methodological viewpoint, their approach stresses the relevance of archaeolinguistic research, a developing field that combines language reconstruction and archaeology as a source of information on human prehistory.

Supported by a team of colleagues from Jilin University, geneticist Yinqui Cui, archaeologists Wei Zhang and Lixin Wang and linguist Martine Robbeets shed a bioarchaeological light on the expansion of Transeurasian languages in the Amur River Basin. Although Northeast China is among the first regions in the world where agriculture developed and is home to the primary dispersals of the Transeurasian languages, its genetic history remains largely unknown. Owing to genetic admixture, it is difficult to reconstruct the migration history of the various ethno-linguistic groups in the Amur Basin on the basis of modern data alone. In this study, the authors try to remedy this situation by retrieving the whole genome and Y chromosome lineages from late Neolithic Honghe individuals in the Middle Amur region. For this purpose, they rely on the analysis of ancient DNA studies as a source of knowledge on prehistoric human migrations and complement their results with insights from archaeology and linguistics. Their genetic analysis reveals that the population of the Amur River Basin has a stable and continuous genetic structure from the Mesolithic up to today. Nevertheless, integrating linguistic and archaeological evidence, they argue that the lack of observable genetic admixture is not contradictory to the expansion of the Transeurasian languages in the Amur River Basin being related to the agricultural development and expansion of the Hongshan culture. They find that millet agriculture played a vital role in the expansion of the population of the West Liao River and Amur regions, which in its turn has contributed to the spread of the Transeurasian languages.

From the Amur region, we then move westward to the Eurasian steppe. Linguist Alexander Savelyev and geneticist Choongwon Jeong examine the origins and history of nomadic groups that ruled over Mongolia and the adjacent parts of southern Siberia and northern China, such as the Xiongnu (ca. 300 BC to 200 AD) and the Rouran (ca. 300–550 AD). Integrating archaeological, linguistic and genetic evidence, they revisit the extent of continuity between Xiongnu–Hun and Rouran–Avar groups and evaluate the proposed association of these groups with speakers of Transeurasian languages such as Turkic, Mongolic and Tungusic languages.

Continuity between the Xiongnu of Inner Asia and the European Huns cannot be tested genetically as no ancient genome from the Hunnic period Carpathian Basin has been reported so far. Although the steppe heritage among the European Huns is very limited from an archaeological perspective, linguistics supports continuity between both groups as they can be associated with ancient varieties of Turkic.

Rouran–Avar continuity is not contradicted from a genetic viewpoint, as both included a component originating in the Eastern steppes, even if this component cannot be specifically identified with the Rouran elites. Linguistically and archaeologically, there is discontinuity in the sense that Rouran can be associated with Mongolic origins, while the European Avars adopted Turkic language and culture.

Triangulating the evidence in this way, this contribution fills important gaps in our understanding of Transeurasian population movements, language dispersals and cultural dynamics on the Eurasian steppe in the Bronze and Iron Ages.

Archaeologist Jangsuk Kim and linguist Jinho Park evaluate the Farming/Language Dispersal Hypothesis in the context of the Korean Peninsula. The authors assess two partly overlapping hypotheses previously proposed by Whitman (2011) and Robbeets (2017a–c) for the spread of the ancestral proto-Japanic and proto-Koreanic language to the Korean Peninsula. Although Whitman and Robbeets agreed on associating the introduction of rice agriculture to the Korean Peninsula around 1300 BC with the dispersal of proto-Japanic, they disagreed about the timing and the cultural association for the advent of proto-Koreanic. Whereas Robbeets suggested that it predated the arrival of proto-Japonic and was associated with the introduction of millet agriculture around 3500 BC, Whitman suggested that it postdated proto-Japonic and was associated with the introduction of bronze daggers around 300 BC.

Whereas Kim and Park support Whitman's and Robbeets’ findings about proto-Japanic, they reject both hypotheses for the advent of proto-Koreanic. On the one hand, they regard Whitman's argument as controversial because the so-called ‘Korean style Bronze dagger’ material culture was very sparsely distributed and connected, in their view, with only very minor migrations. On the other hand, they also reject Robbeets’ hypothesis because the scale of the population migration involved would not have been large enough to support the dispersal of proto-Koreanic and as they fail to detect elements of Northeast Chinese material culture introduced together with millet agriculture.

Alternatively, they consider two scenarios, namely, first, that Proto-Koreanic arrived simultaneously with Proto-Japanic at the beginning of the Mumun period (1300–400 BCE) and second, that the common ancestor Proto-Japano-Koreanic arrived around 1300 BC and separated when it was already present on the peninsula. Admittedly, both scenarios pose serious problems. First, it is difficult to reconcile the unified nature and homogeneous distribution of the rice-farming Mumun culture throughout the Peninsula with two different ethno-linguistic groups who hardly show any signs of having influenced each other in their rice vocabularies. The second hypothesis is contradicted by the absence of rice vocabulary in Proto-Japano-Koreanic and by the early date proposed for the split between both families, predating the introduction of rice farming in 1300 BC.

In light of Nelson et al.'s contribution, discussed above, which suggests that textile technology was introduced together with millet agriculture to the Korean Peninsula, and considering that the linguistic outcome of population migration is less determined by the size of an incoming population than by its cultural impact, the farming/language dispersal of proto-Koreanic may still be open for debate.

Archaeologist Mark Hudson, geneticist Shigeki Nakagome and linguist John Whitman revisit Kazurō Hanihara's (1991) ‘dual structure hypothesis’, which models the modern Japanese as an admixture of Neolithic Jomon populations with Bronze Age Yayoi migrants from the Korean peninsula. Integrating recent research in biological anthropology, historical linguistics and archaeology, the authors investigate whether the biological admixture proposed by Hanihara is supported by recent advances in ancient DNA analysis and how it could have been achieved in social terms.

Whereas the answer to the first question is positive, the authors point out that in the 30 years since Hanihara's article, archaeological research has shifted back the beginning of the Yayoi period to 900 BC, thus supporting a less abrupt and more gradual expansion of Yayoi culture than previously believed. This leads them to posit a more multicultural context of interaction between Jomon and Yayoi populations, involving cultural exchange and language contact between speakers of ancient varieties of Ainu and Japanese.

As such, Hanihara's ‘dual structure hypothesis’ remains influential to date because it provides a foundation to triangulate genetics, archaeology and linguistics as a window into Japanese population history. Moreover, it expands the problem of the ‘origins of the Japanese’ to a wider Transeurasian scale, leaving room for farming/language dispersal across Northeast Asia.

Linguist Elisabeth de Boer, geneticist Melinda Yang, historian Aileen Kawagoe and archaeologist Gina Barnes apply the Farming/Language Dispersal Hypothesis to Japan. They question whether the Japanese language was introduced simultaneously with agriculture on the Japanese islands, paying special attention to the regions of Hokuriku and northern Tohoku in northeastern Japan. More particularly, they examine how Japanese spread to this region, whether its dispersal was indeed associated with agriculture and how it replaced or co-existed with local Jomon languages.

To address these questions, the authors bring together different lines of evidence from genetics, archaeology and linguistics. Even if these disciplines are applied to different time ranges, taken together, they shed light on the transformation of agriculture, language and genome in the Yayoi period (900 BC to AD 250).

In contrast to previous assumptions that the Jomon (14 500–900 BC) were genetically isolated, the authors argue for connections with coastal populations in northern and southern East Asia. They argue for the survival of unadmixed Jomon people into Yayoi times, such as the Epi–Jomon in northeastern Japan, who must have coexisted there with admixed Yayoi people. Archaeology suggests that irrigated rice agriculture was established by Yayoi farmers in Hokuriku and Tohoku as early as 380 BC, at least a century earlier than in the Kanto, Chubu and Tokai regions. At the same time, linguistics shows that certain innovations of phonology and tone systems originated in Izumo and were introduced to the northeast by way of the Japan Sea coast, both to Hokuriku and to Tohoku.

Integrating these observations leads to the hypothesis that the Izumo dialect may have spread with the introduction of rice agriculture by admixed Yayoi peoples in the Middle to Late Yayoi. However, given the relatively low internal diversity and presumed young age of the Tohoku dialects, the authors do not exclude an alternative scenario that attributes the spread of the Izumo dialect to the northeast to later expansions of the mounded tomb culture in the Kofun period (AD 300–645).

Anthropologist Gyaneshwer Chaubey and linguist George van Driem search for patterns of correlation between genetic and linguistic phylogeography with a special focus on Japanese, Korean and the Munda branch of Austroasiatic. Previously it has been proposed that linguistic expansion is sex-biased. Whereas some scholars emphasized ‘the role of maternal transmission of language’ (Nasidze et al., 2006: 671), others stressed the importance of paternal transmission (Poloni et al., 1997). The Munda languages, for instance, represent a clear-cut case of ‘father tongues’ (Chaubey et al., 2011). Mapping ethnolinguistic prehistory on the phylogeography of the Y-chromosomal lineage O, the authors try to answer the question why the father tongue correlation is missing in the case of Japanese and Korean.

Accepting the hypothesis that Japanese is a Transeurasian language, they find that the paternal lineage C2 (M217), which is correlated with Altaic linguistic affinity in Turkic, Mongolic and Tungusic speakers, entered Japan at the beginning of the Early Jomon period (5000–3000BC).

From this observation, they infer that the Japonic language must have entered Japan as early as 5000 BC, predating the introduction of farming by more than 3 millennia. As the Y-chromosomal haplogroup C2 accounts for only 11% of Korean and 6% of Japanese paternal lineages, they further stress that these languages cannot be considered as cases of ‘father tongues’. Their results contradict the prevailing view that proto-Japonic was a farmer language introduced at the beginning of the Yayoi period (Hudson, 1999; Whitman, 2011; Pellard, 2015; Francis-Ratte, 2016; Robbeets et al., 2020), a conclusion supported in this collection by Nelson et al., de Boer et al. (2020) and Hudson et al. (2020).

However, the early introduction of the Northeast Asian paternal lineage C2 may receive an alternative explanation in the light of de Boer and colleagues’ findings. Early gene flow between coastal populations in northern East Asia and Jomon people and the frequency of the alleged ‘Para-Austroasiatic’ O2b lineage in Japan may be explained by gene flow between speakers of Proto-Japonic and Southeast Asian languages at a time before 1500 BC – when rice was added to the agricultural package and Japonic was still spoken in the Shandong–Liaodong interaction sphere (Robbeets, 2017b). In this way, the hypothesis that the founding dispersals of the Japonic family coincided with the spread of agriculture may remain the most parsimonious one.

Archaeologist Junzo Uchiyama, geographic information scientist Christopher Gillam, linguist Alexander Savelyev and geneticist Ning Chao examine population dynamics in the Northern Eurasian Greenbelt (NEG), the northern forest zone stretching from the Japanese Archipelago to Northern Europe. Exploring how the ecological conditions in the NEG impacted human prehistory, they focus on the question of why the Altai–Amur–Japan area repeatedly became the starting point of major events in human prehistory. They observe that this region has been a source region for cultural innovation and human migration, as seen in the development of blade production and ceramic technology around 20,000 years ago, the peopling of the Americas between 18,000 and 15,000 years ago, the development of millet agriculture associated with the origins of the Transeurasian languages around 8,000 years ago and the spread of the complex economy of the Proto-Turkic-speaking community around 2,000 years ago. Taking an interdisciplinary approach, synthesizing environmental archaeology, genetic-geography and linguistics, they propose a possible explanation for this observation from an ecological perspective. They find that the Altai–Amur–Japan region packs diverse biomes of the NEG, such as different kinds of forests, grasslands and freshwater and maritime environments, into a relatively small area. Historically, this allowed people to access diverse resources in different environments with minimum mobility and to adopt mixed subsistence strategies. This advantage turned them into successful innovators and migrators, able to flexibly adapt to changing environments and conditions. From this perspective, the reason for the wide-scale spread of the Transeurasian languages, which is the key subject of this special collection, should not be specifically seen as the result of language/farming dispersal, but could be more generally associated with the mixed subsistence strategies and the successful adaptation to changing environments of early speakers.
